# Communication Strategies for Tick-Borne Disease Prevention Among US Cattle Producers: Quasi-Experimental Study on Message Framing and Source Credibility

**DOI:** 10.2196/77239

**Published:** 2025-12-08

**Authors:** Cheng-Xian Yang, Lauri M Baker, Jessica Czipulis

**Affiliations:** 1 Graduate Institute of Bio-Industry Management, College of Agriculture and Natural Resources, National Chung Hsin University, Taichung City, Taiwan; 2 Department of Agricultural Education and Communication, Center for Public Issues Education in Agriculture and Natural Resources, University of Florida, 1408 Sabal Palm Drive, Level 2, P.O. Box 110126Gainesville, FL, 32611, United States, 1 3522732093; 3 Department of Agricultural Education and Communication, College of Agricultural and Life Sciences, University of Florida, Gainesville, FL, United States

**Keywords:** cattle producers, *Haemaphysalis longicornis*, message framing, quasi-experiment, risk communication, tick prevention

## Abstract

**Background:**

*Haemaphysalis longicornis* (Asian longhorned tick) presents a growing threat to cattle health in the United States, causing anemia, weight loss, and even death. Despite the expanding distribution of this tick and the need for prevention, there is limited research on how to effectively communicate tick-borne disease risks to agricultural communities, particularly cattle producers. Social media represents a potentially impactful channel for risk communication; however, its utility and optimal message design for this population remain insufficiently understood.

**Objective:**

This study aimed to evaluate the effectiveness of various digital communication strategies in influencing US cattle producers’ engagement, risk perceptions, and self-reported preventive intentions regarding tick-borne diseases.

**Methods:**

A quasi-experimental study with a 2×3 factorial design was conducted with 116 cattle producers in the United States. Participants were randomly assigned to view simulated Facebook posts that varied by message source (extension agent vs cattle producer) and message framing (prevention-oriented, fear-based, or neutral). Participants reported their likelihood of engaging with the post (using an emoji reaction, commenting, or sharing), their perceptions (fear and attitudes), and their intention to adopt preventive behaviors. Data were analyzed using descriptive statistics, ANOVA, and Spearman rank-order correlations.

**Results:**

Across all groups, participants showed a clear preference for low-effort engagement, most frequently using emoji reactions rather than commenting or sharing. Descriptive trends suggested that posts from cattle producers, especially those framed as prevention oriented, elicited higher mean engagement. Prevention-oriented messages were also associated with greater concern about tick risks and more favorable attitudes toward prevention. As reflected in the qualitative feedback, fear-based posts were often viewed as exaggerated or less credible. Social media engagement showed moderate correlations with attitudes (ρ=0.52-0.64) and preventive intentions, with attitudes more strongly associated with behavioral intent than fear.

**Conclusions:**

This study provides preliminary evidence that prevention-oriented digital messages, especially when shared by credible sources, may foster more positive attitudes and greater willingness to undertake tick prevention among cattle producers, compared to fear-based or neutral content. The findings highlight the need for balanced, context-rich messaging tailored to the preferences and realities of agricultural audiences. By integrating quantitative and qualitative results, this study informs the design of more effective social media–based interventions for tick-borne disease prevention in agricultural settings.

## Introduction

### Background

*Haemaphysalis longicornis*, also known as the Asian longhorned tick, is originally from East Asia but has established invasive populations in regions such as Australia, New Zealand, and several Pacific islands. This is an invasive species that was first detected in the United States in 2017 and has since been reported in 19 states, particularly in the eastern and southeastern regions [1-5]. The rapid proliferation of *H longicornis* across the United States can be attributed to its broad host range and its ability to reproduce rapidly through a parthenogenetic life cycle, allowing females to reproduce without the need for males [5-9].

*H longicornis* is considered a significant pest, particularly for livestock, where it poses substantial challenges to agricultural productivity and animal health. It is particularly dangerous to the livestock industry because it is a known vector of *Theileria orientalis* Ikeda, a hemoparasite that causes bovine theileriosis, in the United States [2,6,8,10-13]. Heavy infestations of *H longicornis* on cattle can have direct, detrimental impacts on cattle health, including significant blood loss, anemia, reduced production, weight loss, and even death [2,4,5,7,8]. Due to its expanding range and ability to carry pathogens, this tick species is considered a growing concern for the agricultural industry and public health in the United States [1,14].

Effectively managing the risks posed by *H longicornis* to the livestock industry requires comprehensive tick control strategies tailored for cattle producers. Recommended practices include mechanical approaches such as tick surveillance, manual removal, and vegetation management to limit tick habitats, as well as chemical measures like the application of on-animal acaricides or tick repellents [10,12,15,16]. Maintaining a closed herd is also advised to prevent the introduction and spread of ticks among livestock [10]. However, for these preventive strategies to be adopted effectively, raising awareness of tick-borne diseases and the risks they pose is essential. Educational interventions targeted at livestock producers can improve knowledge and awareness and promote the consistent use of proven prevention and control methods [17-19].

Supporting this need, prior research has shown that individuals with higher knowledge levels and greater awareness and concern about tick-borne diseases are more likely to engage in preventive behaviors [20-23]. Nevertheless, a widespread lack of confidence in the effectiveness of tick prevention products such as acaricides and repellents may discourage their use [23]. It has been reported that cattle producers often apply acaricides only occasionally and in response to visible tick presence rather than as part of a consistent, preventive routine [24]. Previous studies recommend that educational efforts address disease transmission routes, tick biology, and validated control strategies to support more systematic management practices. These findings highlight the importance of communication in shaping not only knowledge but also behavior.

Building on this insight, this study approached the issue from a risk communication perspective to better understand how information about tick-borne disease prevention in the context of *H longicornis* is perceived and acted upon by cattle producers. The goal was to inform more effective communication strategies that enhance awareness, improve risk perception, and ultimately encourage the adoption of preventive behaviors among this high-risk population.

### Literature Review and Theoretical Framework

Effective disease communication strategies can enhance public understanding, influence decision-making, and promote behaviors that support disease prevention [25-27]. To ensure maximum impact, communicators should customize their strategies to fit the specific behaviors and needs of target populations, including those at risk for tick-borne diseases, and account for varying contexts [28-30]. For example, digital technologies and online platforms have rapidly expanded the ways in which risk information can be delivered and received.

The rise of social media has revolutionized global communication, especially in fields like agriculture, where these platforms serve as powerful tools for disseminating technology and information [31]. Social media, with its potential for real-time updates and interactive engagement, is suited for risk communication, including that for tick-borne diseases [32-34]. These features allow communicators to effectively engage with the public, fostering a better understanding and management of tick risks [19,35]. Gupta et al [17] further indicated that social media appears to be an effective communication channel for spreading knowledge about tick-borne diseases. Platforms such as Facebook (Meta Platforms, Inc), X (formerly Twitter; X Corp), and YouTube (Google Inc) have become essential channels for sharing scientific information, making them indispensable for communicating risks [36,37].

The interactive nature of social media promotes risk perception and informed decision-making during disease outbreaks. Previous studies have pointed out that social media content about vector-borne diseases is positively related to users’ awareness and knowledge of the risk and preventive actions [38-40]. Although previous research suggests that cattle producers in the United States generally have a lower preference for and lower trust in social media [41], it also highlights that information shared through social media by credible sources such as peer cattle producers and beef industry organizations is often considered reliable [42]. Therefore, while social media may not be the preferred communication tool for this demographic, its importance in reaching broader audiences and conveying critical disease prevention messages cannot be underestimated.

In the context of US cattle producers, extension agents and peer cattle producers represent two of the most influential yet contrasting sources in terms of credibility and practical relevance. Extension agents have institutional expertise and are scientific authority figures. They serve as formal representatives of land-grant universities or government agricultural agencies. Their credibility stems from professional training, research-based knowledge, and access to current scientific information. Many producers view them as reliable and unbiased sources of practical advice. Effective communication by extension agents, especially when combined with interpersonal skills and an understanding of producer needs, can enhance the adoption of innovations and best practices [43-45]. In contrast, peer cattle producers are trusted for their practical experience and shared challenges. Producers see peers as credible because they have faced similar decisions in the same environment. Peer learning and observation strongly influence behavioral change, as agricultural producers trust advice from those living comparable realities [46,47]. Unlike extension agents, peers shape behavior through social connections and proven local success, offering a grassroots influence that complements expert-driven approaches.

Additionally, designing communication content that resonates with the target audience enhances the effectiveness of risk communication. Framing theory is often used to explain how the presentation of information through communication channels influences how audiences interpret and respond to a message. A frame highlights certain aspects of an issue while downplaying others, thus shaping the audience’s perception of the topic [48]. In the context of disease communication, gain-framed messages focus on prevention and present outcomes as clear and low-risk, conveying the idea that the disease is manageable. On the other hand, loss-framed messages, which emphasize the potential health risks, tend to communicate greater uncertainty and evoke stronger negative emotions such as fear [19,49-51]. Gain-framed appeals, which highlight the advantages of compliance, have statistically higher engagement rates and are more persuasive than loss-framed appeals, but the difference in effectiveness is quite small [52,53]. While this finding is supported by several studies [49,54], other research has demonstrated that framing messages around the severity of risk can also be effective in encouraging preventive actions [50,55]. Li et al [56] found that social media posts combining both positive and negative content generate more comments and reactions than those with purely negative content.

### Purpose

This exploratory study aimed to identify effective social media communication strategies for cattle producers in the United States and to explore the factors related to their risk-preventive intentions regarding the impact of *H longicornis* on the livestock industry. Adoption of personal preventive actions varies depending on individuals’ perceptions of tick-borne disease risks and their demographic characteristics. Therefore, targeted health promotion communication is essential for high-risk groups exposed to ticks. This area remains underresearched, and communicators should focus on ensuring that these at-risk populations adopt necessary prevention behaviors [15-17,19,30,57]. The following research questions were developed to align with the research objectives.

1. Which message framing is observed to engage cattle producers more when presenting the risks of *H longicornis*?
2. How do cattle producers perceive different types of message framing regarding fear, attitudes, and consideration of preventive behavioral intentions regarding the risks of *H longicornis*?
3. What are the associations among social engagement, perceptions, and preventive behavioral intentions to identify the key variables in cattle producers’ responses?

## Methods

### Population and Sample

This quantitative study surveyed cattle producers in the United States using an online survey distributed via Qualtrics. Given the difficulty in reaching the target population, both online and offline methods were used to gather responses through convenience sampling. At the annual convention of the National Cattlemen’s Beef Association (NCBA) in February 2024 in Orlando, Florida, we collected data directly from cattle producers. They were asked to complete the online questionnaire using iPads (Apple) and QR codes, resulting in 68 responses. Additionally, from May to June 2024, we collected 48 more responses through Facebook ads and advertisements in cattle magazines. A total of 116 valid responses were obtained in this study. Nonprobability sampling was adopted as a suitable method for making population estimates in this context because it allows targeting specific, hard-to-reach populations while maintaining a broad outreach, even though it does not involve random selection [58].

### Research Design and Instrument

At the beginning of the questionnaire, we asked participants to identify the primary class of commodity they grow or produce. If “cattle” was not selected as one of the primary commodities, the respondent was excluded from the study to ensure that the collected sample aligned with our target population. Variables such as location and annual sales for the agricultural operation were also collected to gather comprehensive background information on the participants. Additionally, a pretest question, which required a response on a 5-point Likert scale, was asked to understand the respondent’s support for tick risk education.

The study used a quasi-experimental approach. To ensure complete and transparent reporting, this study adhered to the Checklist for Reporting Results of Internet E-Surveys (CHERRIES) [59], appropriate for quasi-experimental studies and web-based interventions. Specifically, we followed the recommendations for reporting internet-based surveys to ensure clear description of the participant recruitment and allocation procedures and data collection methods. These guidelines supported consistent and detailed reporting of our study methods and helped ensure that the study is interpretable and reproducible by others.

A 2×3 factorial design was implemented to examine the influence of message source (extension agent versus cattle producer) and message framing (prevention-oriented, fear-based, or neutral) on producer perceptions and behavioral intentions related to tick prevention. This design created 6 experimental conditions, with each participant randomly assigned to view 1 message from each source, totaling 2 messages per participant. Random assignment was used to minimize selection bias and balance potential confounders across groups.

To guage cattle producers’ perceptions of social media posts, this study developed a survey instrument focused on creating realistic simulated Facebook posts. Zeoob, an online educational tool that can generate simulated social media posts [60], was chosen for this purpose. Six posts were created, all related to *H longicornis*, and attributed to 2 personas—an extension agent and a cattle producer. Each persona shared 3 posts with different content. The content framing varied from a prevention-oriented message, which was gain framed (M1: “It is crucial to spray your pastures and animals to prevent ticks from infecting the herd.”), to a fear-based message, which was loss framed (M2: “Watch out! Cow-killing ticks are spreading across the U.S.”), and included a middle-ground approach (M3: “You don’t want to learn the hard way. Entire herds across the U.S. have been lost already.”).

Simulated posts were presented via the online survey platform. We excluded responses that were completed too quickly to ensure that respondents had sufficient exposure time to view each post. Participants could not revisit previous messages. Participants could view only the posts assigned to their experimental condition and had no opportunity to view posts from other subgroups.

The survey questions assessed participants’ likelihood of social engagement after viewing the social media post. Participants were asked how they would react, including choosing an emoji, commenting, or sharing the post. The likelihood of engagement was measured on a 5-point Likert scale (1=unlikely, 2=somewhat likely, 3=likely, 4=very likely, and 5=extremely likely). If participants indicated that they were at least somewhat likely to react by liking the post, they were further asked to select which emoji they might use. These emojis, modeled after those on Facebook, included 7 options such as like, love, and sad.

We also assessed their perceptions of the post, including their fear of *H longicornis* and the diseases it spreads as well as their attitudes supporting proactive preventive intentions and raising awareness about tick-related risks. To assess the participants’ preventive behavioral intentions in response to tick-borne diseases, we focused on 2 key measures: removing ticks through surveillance and spraying cattle with on-animal acaricides or tick repellents [10]. Each measure was assessed with 2 questions, all answered on a 5-point Likert scale (1=strongly disagree to 5=strongly agree).

**Figure 1.**
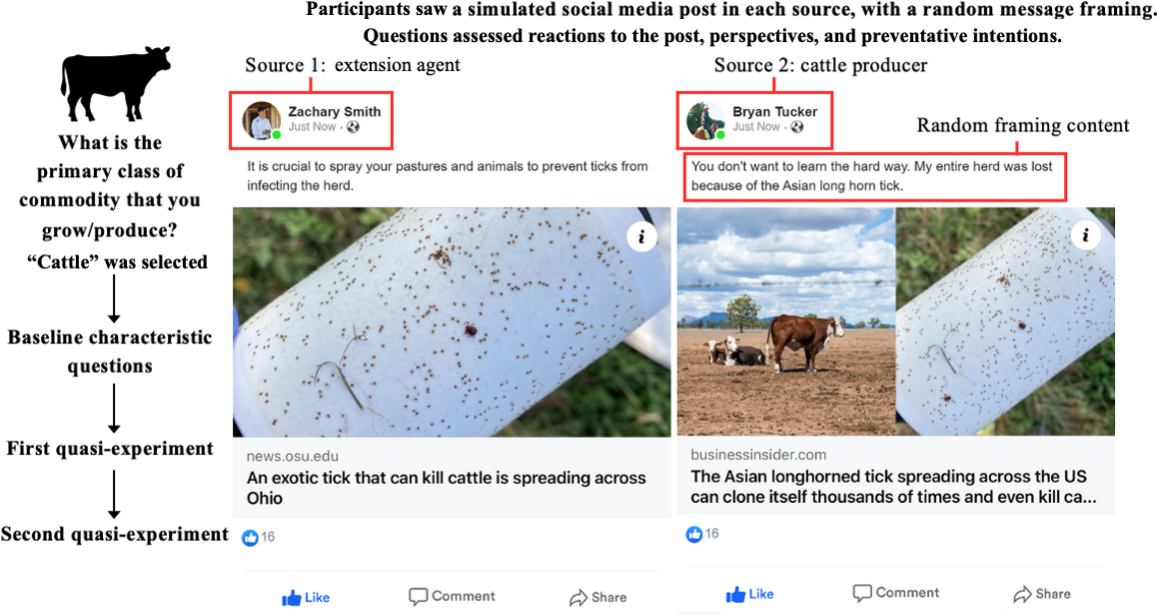
Research process and illustration of the quasi-experiment.

Moreover, to enrich our findings, we included open-ended questions to capture the participants’ thoughts and reflections on the simulated social media posts viewed in their assigned experimental condition. Each respondent answered up to 28 questions, including 6 about their baseline characteristics as well as 2 sets of quasi-experimental questions (11 questions each). Figure 1 depicts the research process and the simulated social media posts.

### Data Analysis

SPSS software (version 29.0; IBM Corp) was used to analyze the quantitative data. The random assignment method was expected to distribute potential confounders evenly. We conducted ANOVA tests to compare baseline demographic variables and pretest attitudes across the randomized groups, using group assignment as the independent variable. All comparisons were nonsignificant, indicating that the groups did not differ in these baseline measures. To answer our first and second research questions, descriptive statistics were first used to explain how participants responded to different messages. ANOVA tests with post hoc comparisons were completed using the Scheffe correction. For the third research question, Spearman rank-order correlations were used to examine the associations between their social engagement on social media, perceptions of the post, and reported preventive intentions, given the ordinal nature of the Likert-type items.

Qualitative data were analyzed using a thematic analysis approach. First, all responses were read in full to ensure familiarization with the content. Initial codes that captured distinct concepts or sentiments were identified. Then, through an iterative process, similar codes were grouped into broader themes that illustrated cattle producers’ perspectives on message tone, credibility, perceived relevance, and barriers or motivators to engagement. Representative quotes were selected to illustrate each major theme. This procedure ensured a rigorous and transparent analysis of qualitative inputs, complementing the quantitative survey results.

### Ethical Considerations

This study was reviewed and approved by the Institutional Review Board at the University of Florida (IRB#202201562). Written informed consent was obtained electronically from each participant prior to their participation. Participants were briefed about the study objective, procedures, voluntary nature of participation, and the right to decline or withdraw at any time without penalty. All data collected were anonymized before analysis. Participants were given a tick removal kit for their participation in the study. The value of each kit was under US $2.

## Results

### Descriptive Statistics of Agricultural Operation Characteristics

Due to convenience sampling, most of the respondents came from southeastern states in the United States, such as Florida (30/116, 25.9%), Tennessee (20/116, 17.2%), and Kentucky (14/116, 12.1%). The agricultural operations of most cattle producers (98/116, 84.5%) were located in rural areas. Approximately half of the respondents (55/116, 47.4%) had annual sales of less than US $50,000, followed by those with sales between US $50,001 and US $150,000 (24/116, 20.7%). These figures reflect the national data from the 2022 Census of Agriculture, which reported an average annual agricultural income of US $79,790 [61]. Regarding their role in agriculture, 89 participants (76.7%) identified as owner or operator. Detailed characteristics of the sample are shown in Table 1.

**Table 1.** Characteristics of the sample (N=116).

Characteristic	Participants, n (%)
State of origin	
Florida	30 (25.9)
Tennessee	20 (17.2)
Kentucky	14 (12.1)
Texas	9 (7.8)
Virginia	9 (7.8)
Georgia	8 (6.9)
Other	26 (22.4)
Agricultural operation location	
Rural area	98 (84.5)
Urban or suburban area outside the city limits	14 (12.1)
Urban or suburban area inside the city limits	4 (3.4)
Agricultural operation annual sales	
Less than US $50,000 in gross annual sales	55 (47.4)
Between US $50,001 and US $150,000 in gross annual sales	24 (20.7)
Between US $150,001 and US $249,999 in gross annual sales	12 (10.3)
Between US $250,000 and US $500,000 in gross annual sales	13 (11.2)
More than US $500,000 in gross annual sales	12 (10.3)
Role in agriculture	
Owner/operator	89 (76.7)
Hired labor	9 (7.8)
Consumer	4 (3.4)
Other	14 (12.1)

### Social Engagement on Social Media

To answer the first research question, in the quasi-experiment, we assessed cattle producers’ self-reported engagement with different simulated social media posts, specifically through choosing an emoji, commenting, and sharing the post. While the ANOVA did not reveal statistically significant differences across the 6 combinations of message source and message type, we observed several noteworthy descriptive patterns in the engagement data.

Overall, the results indicated that cattle producers are most likely to engage with posts through emoji reactions compared to commenting and sharing. Among the various emojis available on Facebook, the “Like” emoji remained the most frequently used in all experimental conditions, even compared to newer emojis. Commenting was generally less likely across all combinations of message source and type. The mean scores for commenting were consistently lower than those for emoji reactions and sharing, suggesting that while cattle producers may react to or share posts, they are less likely to engage in discussions or leave comments on these posts.

Further examination showed some variation in the mean engagement scores across the combinations of message source and type and highlighted some trends. For instance, the highest average likelihood score for emoji engagement (mean 2.89, SD 1.30) was observed for the cattle producer × M1 (cattle producer, prevention-oriented message) group, while the lowest (mean 2.38, SD 1.33) was observed for the extension agent × M2 (extension agent, fear-based message) group. Similar descriptive differences were noted for the sharing and commenting behaviors. However, it is important to note that these observed patterns did not reach statistical significance in this analysis and thus should be interpreted with caution as preliminary trends rather than as definitive group differences. Table 2 presents the descriptive results for social engagement across all experimental conditions.

**Table 2.** Likelihood of social engagement with simulated Facebook posts among participants in 6 experimental conditions.

Type of engagement	Likelihood score^a^, mean (SD)					
	EA^b^ × M1^c^ (n=40)	EA × M2^d^ (n=34)	EA × M3^e^ (n=40)	CP^f^ × M1 (n=36)	CP × M2 (n=32)	CP × M3 (n=38)
Emoji	2.60 (1.37)	2.38 (1.33)	2.81 (1.37)	2.89 (1.30)	2.42 (1.17)	2.76 (1.46)
Comment	2.05 (1.36)	1.62 (1.13)	1.86 (1.24)	2.00 (1.29)	1.72 (1.05)	2.03 (1.40)
Share	2.33 (1.39)	2.35 (1.43)	2.30 (1.52)	2.42 (1.42)	2.03 (1.18)	2.63 (1.53)

a Likelihood was rated on a 5-point Likert scale.

b EA: extension agent.

c M1: prevention-oriented message.

d M2: neutral message.

e M3: fear-based message.

f CP: cattle producer.

### Perceptions of Simulated Social Media Posts and Preventive Intentions

To answer the second research question, we assessed several important dimensions, including participants’ fear and concern about tick-related risks, attitudes supporting preventive behaviors and local awareness, and their self-reported intentions to take preventive actions following exposure to the social media posts. Across these outcomes, while ANOVA results did not indicate significant differences between groups, we identified some descriptive patterns that may offer preliminary insights for future studies.

Regarding the respondent’s perception of the post, it was observed that the source of the message alone did not make a difference. However, when combined with the content of the message, distinct trends began to emerge. Specifically, when either an extension agent or a cattle producer used a prevention-oriented message to communicate the risks of *H longicornis* to the cattle, participants expressed higher levels of concern about tick risks. They also demonstrated more positive attitudes toward managing the risks associated with ticks. Conversely, when a fear-based message was employed as the communication strategy, it led to lower perceptions of post credibility or effectiveness.

Regarding responses related to preventive intentions, this study observed distinct patterns across different messaging sources and types. An analysis of responses to messages from an extension agent showed that the neutral message proved effective in prompting preventive intentions. For example, the extension agent × M2 condition had a relatively high mean score for examining gear and cattle for ticks (mean 3.94, SD 0.75) and using tick repellents (mean 3.88, SD 1.02). However, when cattle producers were the source of the message, the data revealed somewhat mixed outcomes. The prevention-oriented message had a relatively high mean score for encouraging cattle producers to examine gear and cattle for ticks as a precaution (mean 3.94, SD 0.89), but this communication strategy was less effective in promoting the use of tick repellents to avoid tick bites (mean 3.62, SD 1.23). Table 3 shows the perceptions of the posts and preventive behavioral intentions among participants in various experimental conditions.

**Table 3.** Perceptions of simulated Facebook posts and preventive intentions among participants in 6 experimental conditions.

Statement	Rating^a^, mean (SD)					
	EA^b^ × M1^c^ (n=39)	EA × M2^d^ (n=33)	EA × M3^e^ (n=40)	CP^f^ × M1 (n=34)	CP × M2 (n=33)	CP × M3 (n=34)
Fear						
Worries about cattle health	3.77 (0.90)	3.52 (1.09)	3.60 (0.84)	3.85 (0.93)	3.73 (0.67)	3.50 (1.02)
Fear of tick risks	3.28 (0.92)	3.09 (0.95)	3.22 (0.89)	3.53 (0.93)	3.39 (0.75)	3.32 (1.07)
Attitude						
Attitude toward educating the community about tick risks	4.08 (0.74)	3.88 (1.17)	4.07 (0.94)	4.09 (0.90)	4.00 (0.71)	3.91 (0.90)
Attitude toward regular checks for ticks in the surroundings	4.08 (0.87)	4.15 (0.83)	3.68 (1.07)	3.97 (0.87)	3.94 (0.75)	3.79 (0.85)
Preventive intentions						
Regular examination of gear and cattle for ticks	3.77 (0.87)	3.94 (0.75)	3.50 (1.13)	3.94 (0.89)	3.70 (0.81)	3.82 (0.97)
Use of tick repellents to avoid tick bites	3.67 (0.90)	3.88 (1.02)	3.67 (1.07)	3.62 (1.23)	3.82 (0.81)	3.97 (0.83)

a Statements were rated on a 5-point Likert scale.

b EA: extension agent.

c M1: prevention-oriented message.

d M2: neutral message.

e M3: fear-based message.

f CP: cattle producer.

### Open-Ended Responses From Cattle Producers

To interpret and supplement the quantitative results, we further asked participants for their opinions on the simulated social media posts to gain deeper insights into their thoughts on the experimental conditions. Some responses were brief affirmations, such as “Good job” or “Great info.” These responses suggest that certain producers viewed the posts as useful and well presented, even if the messages did not always prompt further engagement or detailed feedback. Conversely, some participants offered minimal or neutral input, with responses such as “No” or “N/A.” Such replies indicate that not all respondents felt strongly about the content or were motivated to provide additional commentary.

After excluding brief or ambiguous responses, thematic analysis was conducted to identify distinct concepts emerging from the qualitative data. This approach yielded 3 dominant themes. The most frequently occurring theme was criticism of sensationalism and scare tactics. Respondents expressed concern over the use of alarming or exaggerated language in the social media posts. For instance, comments such as “Seems like a fear tactic instead of informational,” “Over exaggerated,” and “Sounds like a scare tactic by PETA [People for the Ethical Treatment of Animals].” All comments about the content being exaggerated were about posts with a fear-based or neutral message, with slightly more comments directed at posts with fear-based messages. This underscores the perception that fear-based messaging may undermine credibility, potentially resulting in skepticism or disengagement among the target audience. While some participants acknowledged that such messages were effective in capturing attention, several emphasized that communication should prioritize education over sensationalism, as reflected in the following comment: “The post should be more educational rather than shocking.”

Another closely related but different theme was social media skepticism and fatigue. Under this theme, participants expressed doubt not only about the content of the posts but also about the reliability and intentions behind social media communication in general. Comments such as “[It was] too much of a scare tactic to get someone to click the link” and “Less clickbait, obviously untrue” reflect broader concerns about the prevalence of clickbait in social media environments. Moreover, comments such as “I would look for verification on something other than Facebook before I reacted” suggest a distrust of information on social media. The primary distinction between this theme and the previous one lies in the focus: while criticism of sensationalism centers on the content and style of the specific posts, social media skepticism and fatigue reflect a deeper mistrust toward the platform itself and weariness with manipulative online communication strategies.

Additionally, a prominent theme was the desire for more detailed and contextual information. Several participants explicitly stated the need for “more information” or greater context, as exemplified by comments such as “Could use more information in general,” “I would like it to elaborate more,” and “I think more context should be included!” These responses may suggest that while social media posts can raise initial awareness, producers are looking for more substantive and actionable content that offers practical guidance on tick risks and prevention strategies. The call for additional information likely stems from the perception that the information in the simulated posts lacked sufficient detail or depth to fully inform decision-making or address producers’ informational needs. Additionally, some respondents highlighted the importance of factual accuracy, expressing a preference for information backed by evidence, through responses such as “Would react to see back up verification that it was real.” All comments under the theme of desire for more detailed and contextual information were directed at posts with fear-based or neutral messages, with the latter receiving more comments.

### Associations Between Social Engagement, Perceptions, and Preventive Intentions

The analysis of social engagement, which included using emoji reactions, commenting, and sharing, revealed associations with risk perceptions, attitudes, and reported preventive intentions. Emoji reactions were highly correlated with both sharing (ρ=0.83, *P*<.001) and commenting (ρ=0.80, *P*<.001), suggesting that participants who engaged with posts through emojis were also more inclined to engage further by sharing or commenting. Furthermore, social engagement exhibited meaningful associations with perceptions and preventive actions. Participants’ interactions with social media posts showed low to moderate correlations with their perceptions and subsequent behavioral intentions. For instance, those who were more willing to share the post tended to have moderate concerns about the health risks ticks pose to their cattle (ρ=0.32, *P* <.001) and were also more likely to engage in preventive intentions such as regularly examining their gear and cattle for ticks (ρ=0.25, *P*<.001) and using tick repellents (ρ=0.29, *P*<.001).

The results also indicated that fears and attitudes toward managing the risks associated with ticks had a positive association with preventive intentions. The association between fear and preventive intentions was low to moderate. For instance, producers’ worries about cattle health because of ticks were moderately correlated with regular examination of gear and cattle for ticks (ρ=0.34, *P*<.001) and the use of tick repellents to avoid tick bites (ρ=0.33, *P*<.001). On the other hand, attitudes formed after viewing the posts were closely linked to the likelihood of engaging in preventive intentions against tick-borne risks, showing substantial to very strong associations (ρ=0.52‐0.64). All correlations reported in this study were positive. While these correlations point to meaningful links between risk perception, engagement, and behavioral intentions, it is important to emphasize that given the observational design, these associations do not imply prediction or causation. Table 4 shows the associations between social engagement, perceptions, and preventive intentions among the experimental conditions.

**Table 4.** Associations^a^ between various aspects of social engagement, perceptions, and preventive intentions.

	Emoji use	Comment	Share	Worries about cattle health	Fear of tick risks	Attitude toward educating the community about tick risks	Attitude toward regular checks for ticks in the surroundings	Regular examination of gear and cattle for ticks	Use of tick repellents to avoid tick bites
Emoji use, ρ	1.00	0.80^b^	0.83^b^	0.27^b^	0.35^b^	0.13	0.08	0.15^c^	0.16^d^
Comment, ρ	0.80^b^	1.00	0.79^b^	0.22^b^	0.30^b^	0.10	0.10	0.20^b^	0.21^b^
Share, ρ	0.83^b^	0.79^b^	1.00	0.32^b^	0.37^b^	0.18^b^	0.13	0.25^b^	0.29^b^
Worries about cattle health, ρ	0.27^b^	0.22^b^	0.32^b^	1.00	0.57^b^	0.46^b^	0.38^b^	0.34^b^	0.33^b^
Fear of tick risks, ρ	0.35^b^	0.30^b^	0.37^b^	0.57^b^	1.00	0.32^b^	0.32^b^	0.20^b^	0.27^b^
Attitude toward educating the community about tick risks, ρ	0.13	0.10	0.18^b^	0.46^b^	0.32^b^	1.00	0.54^b^	0.53^b^	0.53^b^
Attitude toward regular checks for ticks in the surroundings, ρ	0.08	0.10	0.13	0.38^b^	0.32^b^	0.54^b^	1.00	0.64^b^	0.52^b^
Regular examination of gear and cattle for ticks, ρ	0.15^c^	0.20^b^	0.25^b^	0.34^b^	0.20^b^	0.53^b^	0.64^b^	1.00	0.58^b^
Use of tick repellents to avoid tick bites, ρ	0.16^d^	0.21^b^	0.29^b^	0.33^b^	0.27^b^	0.53^b^	0.52^b^	0.58^b^	1.00

a Strength of the association is indicated by the value of the correlation coefficient [62]: 0.01-0.09, negligible; 0.10‐0.29, low; 0.30‐0.49, moderate; 0.50‐0.69, substantial; >0.70, very strong.

b *P*<.001.

c *P*=.03.

d *P*=.02.

## Discussion

This study provides a clearer understanding of the effectiveness of digital risk communication strategies among cattle producers, specifically regarding social media engagement, risk perception, and preventive intentions in response to messages about *H longicornis*. While some observed differences did not reach statistical significance in our quasi-experimental study, several noteworthy descriptive trends and participant insights emerged from the qualitative data, providing valuable direction for both future research and practical science communication. These findings fill a gap in research on the social media health education interventions on people’s perceptions and preventive intentions [16].

First, various message frames under different conditions were analyzed to better inform communication strategies targeting cattle producers in the United States regarding the health risks posed by *H longicornis* to their livestock. Results showed that cattle producers were more likely to react to posts using emojis than to share or comment, suggesting a preference for low-effort engagement when processing tick-related information. This is consistent with previous digital communication research, indicating that while such responses can enhance message visibility [63], they may not fully reflect deeper cognitive engagement. The “Like” emerged as the most frequently used reaction across all experimental conditions. This aligns with prior research suggesting that clicking the “Like” button is often a cognitively automatic response [63,64]. Users may favor it because it requires minimal mental effort and serves as a default expression of acknowledgment or general approval without necessitating deeper emotional processing or critical engagement.

Contrary to the findings of O’Keefe and Jensen [53], we did not observe statistically significant differences in social media engagement rates between prevention-oriented and fear-based messages, nor did gain-framed messages lead to higher engagement [56]. This result aligns with the findings of Yang et al [19], who observed no clear distinction in social media engagement when tick risks were communicated using either gain or loss framing. Nevertheless, both gain- and loss-framed messages showed a trend of higher levels of social engagement than neutral messages communicating the risks associated with *H longicornis*, regardless of whether the message source was an extension agent or a fellow cattle producer. This contrasts with the findings of Li et al [56], which reported that posts with a middle-ground approach containing both positive and negative content generated higher social engagement. We infer that this descriptive difference may be due to the special nature of the case. Agricultural risks, which often directly affect livestock and livelihoods, may prompt cattle producers to respond more strongly to content that emphasizes these risks than to content that presents risks mildly. This finding highlights that emotionally charged and prevention-oriented content may attract more attention than neutral content, although these differences were not statistically significant in this sample. Despite this finding, qualitative data revealed that some participants have a distrust of the social media platform itself and experienced information fatigue, which in turn affected their responses to the messages.

The results also revealed descriptive patterns suggesting associations between different communication strategies and cattle producers’ perceptions of *H longicornis* risks. Although the message source alone did not appear to substantially affect perceptions, the combination of source and content framing showed some preliminary trends. Specifically, prevention-oriented (gain-framed) messages, whether delivered by an extension agent or fellow cattle producer, were associated with higher levels of concern about tick risks and more positive attitudes toward community education and routine livestock checks. This observation is generally consistent with previous research on prospect theory [49,51,54], which indicates that gain-framed information tends to be more persuasive when promoting actionable outcomes. In this study, the prevention-oriented messages highlighted specific actions that could mitigate risks, potentially supporting the positive attitudinal responses.

Conversely, fear-based (loss-framed) messaging tended to elicit lower perceptions of post credibility or effectiveness. Our qualitative findings further enrich the understanding of communication effectiveness. Some cattle producers reported being less inclined to engage with or trust posts they viewed as sensationalized and did not prefer excessive use of clickbait. Despite acknowledging that emotionally charged language draws attention, several participants cautioned that such approaches can undermine trust or provoke skepticism, echoing broader findings in health communication that fear-based messaging, while memorable, may backfire among certain segments of the population [29,40,52,65,66]. These insights provide actionable guidance that effective digital outreach should balance the need to capture attention with the delivery of accurate, context-rich information that empowers rather than alarms the audience [31,33].

With respect to reported preventive intentions, differences among message types were subtle and did not reach statistical significance. Descriptively, neutral messaging from an extension agent appeared to be relatively more effective in encouraging intentions to undertake both mechanical and chemical tick prevention measures. In contrast, fear-based messaging was associated with a trend of lower engagement in preventive intentions. Notably, prevention-oriented messages from cattle producers were more commonly associated with precautionary behavioral intentions, such as checking gear and cattle for ticks. However, fewer participants reported using more intensive measures, such as tick repellents, following these messages. These trends suggest that while producers may rely on peer recommendations for certain preventive practices [46,47], additional reinforcement or information may be needed to encourage the use of more comprehensive preventive methods such as chemical treatments.

For the last research question, the associations among social engagement, perceptions, and preventive intentions were examined. First, it was found that participants who engaged with posts showed low to moderate correlations with their perceptions, including both their fear of tick-borne diseases and attitudes toward managing tick-related risks. This suggests that as cattle producers develop stronger perceptions of the issue, they are more inclined to interact with related posts, which in turn may amplify their resonance with the content. Furthermore, the study revealed that higher social engagement was also associated with increased implementation of both mechanical and chemical tick prevention measures. This implies the effectiveness of social media not only in engaging the public [32,34] but also in disseminating information about tick-borne diseases [17,19,35]. As this is a cross-sectional study, we cannot infer a direct causal relationship between social media interaction and cattle producers’ perceptions and preventive intentions. The findings of this study present a potential avenue to be explored in greater depth in future research.

Additionally, the study revealed moderate associations between participants’ fear of ticks harming cattle and their intentions to regularly inspect the cattle and gear or apply tick repellents. This is consistent with Olechnowicz et al [57], who highlight the motivating influence of emotional discomfort caused by ticks, as well as with other research showing that increased awareness and concern about tick-borne diseases can promote preventive practices [20-22]. These findings suggest that tick prevention messaging may benefit from incorporating emotional appeals so long as messages remain accurate and clear. On the basis of framing theory [19,51] and our results, we recommend pairing risk-based messages with practical, actionable strategies that empower producers, rather than relying solely on fear. When combined with evidence-based control recommendations [23,24], such an approach is more likely to motivate cattle producers to adopt systematic and sustained tick prevention practices.

This exploratory study has certain limitations. The sample—primarily drawn from the southeastern United States—was modest in size, and baseline participant characteristics were not fully assessed, which may limit the generalizability of the findings. However, the combination of quantitative and qualitative results yields practical recommendations for those seeking to improve digital health risk communication in agricultural sectors. We recommend using larger, more diverse samples and assessing more baseline characteristics in future research, to validate these findings across different regions and agricultural contexts and to explore the potential differences in risk perception and preventive behaviors across diverse agricultural populations.

In the future, communicators should consider the challenge of providing sufficient and practical information about tick-borne diseases within the constraints of brief social media posts. While concise, visually engaging messages are necessary, they must also convey credibility and substance in order to build trust and motivate action. There is no single framing or strategy that works for all target audiences, which makes it an ongoing challenge to effectively communicate the risks of ticks and tick-borne diseases within a limited number of characters in a post while also encouraging the audience to seek more information or take preventive actions [28]. These challenges are important areas for further exploration in future research.

## Abbreviations

CHERRIES: Checklist for Reporting Results of Internet E-Surveys
NCBA: National Cattlemen’s Beef Association
PETA: People for the Ethical Treatment of Animals

## References

[1] (2025). Where ticks live. Centers for Disease Control and Prevention.

[2] Egizi A, Bulaga-Seraphin L, Alt E (2020). First glimpse into the origin and spread of the Asian longhorned tick, *Haemaphysalis longicornis*, in the United States. Zoonoses Public Health.

[3] Rainey T, Occi JL, Robbins RG, Egizi A (2018). Discovery of *Haemaphysalis longicornis* (Ixodida: Ixodidae) parasitizing a sheep in New Jersey, United States. J Med Entomol.

[4] (2024). The longhorned tick: what you need to know story map. Animal and Plant Health Inspection Service - USDA.

[5] (2024). Monitoring Haemaphysalis longicornis, the Asian longhorned tick, populations in the United States. https://www.aphis.usda.gov/sites/default/files/monitoring-h-longicornis-plan-final-january-2024.pdf.

[6] Beard CB, Occi J, Bonilla DL (2018). Multistate infestation with the exotic disease-vector tick *Haemaphysalis longicornis* - United States, August 2017-September 2018. MMWR Morb Mortal Wkly Rep.

[7] Heath ACG (2016). Biology, ecology and distribution of the tick, *Haemaphysalis longicornis* Neumann (Acari: Ixodidae) in New Zealand. N Z Vet J.

[8] Schappach BL, Krell RK, Hornbostel VL, Connally NP (2020). Exotic *Haemaphysalis longicornis* (Acari: Ixodidae) in the United States: biology, ecology, and strategies for management. J Integr Pest Manag.

[9] Tufts DM, Goodman LB, Benedict MC, Davis AD, VanAcker MC, Diuk-Wasser M (2021). Association of the invasive *Haemaphysalis longicornis* tick with vertebrate hosts, other native tick vectors, and tick-borne pathogens in New York City, USA. Int J Parasitol.

[10] Butler RA, Trout Fryxell RT (2023). Management of *Haemaphysalis longicornis* (Acari: Ixodidae) on a cow-calf farm in East Tennessee, USA. J Med Entomol.

[11] Dinkel KD, Herndon DR, Noh SM (2021). A U.S. isolate of *Theileria orientalis*, Ikeda genotype, is transmitted to cattle by the invasive Asian longhorned tick, *Haemaphysalis longicornis*. Parasit Vectors.

[12] Eleftheriou A, Beckett J, Bai N, Pesapane R (2023). An established population of Asian longhorned ticks (Acari: Ixodidae) in Ohio, USA. J Med Entomol.

[13] Thompson AT, White S, Shaw D (2020). *Theileria orientalis* Ikeda in host-seeking *Haemaphysalis longicornis* in Virginia, U.S.A. Ticks Tick Borne Dis.

[14] Beard CB, Visser SN, Petersen LR (2019). The need for a national strategy to address vector-borne disease threats in the United States. J Med Entomol.

[15] de la Fuente J, Estrada-Peña A, Rafael M (2023). Perception of ticks and tick-borne diseases worldwide. Pathogens.

[16] Eisen L (2022). Personal protection measures to prevent tick bites in the United States: knowledge gaps, challenges, and opportunities. Ticks Tick Borne Dis.

[17] Gupta S, Eggers P, Arana A (2018). Knowledge and preventive behaviors towards tick-borne diseases in Delaware. Ticks Tick Borne Dis.

[18] Herdiana H, Sari JFK, Whittaker M (2018). Intersectoral collaboration for the prevention and control of vector borne diseases to support the implementation of a global strategy: a systematic review. PLoS One.

[19] Yang CX, Baker LM, McLeod-Morin A (2024). Tweet tweet tick: a quantitative content analysis of risk communication about ticks on Twitter. Front Commun.

[20] Cuadera MKQ, Mader EM, Safi AG, Harrington LC (2023). Knowledge, attitudes, and practices for tick bite prevention and tick control among residents of Long Island, New York, USA. Ticks Tick Borne Dis.

[21] Hansen MF, Sørensen PK, Sørensen AE, Krogfelt KA (2023). Can protection motivation theory predict protective behavior against ticks?. BMC Public Health.

[22] Vasić A, Bjekić J, Veinović G (2022). Knowledge, attitudes, and practices on tick-borne encephalitis virus and tick-borne diseases within professionally tick-exposed persons, health care workers, and general population in Serbia: a questionnaire-based study. Int J Environ Res Public Health.

[23] Zöldi V, Turunen T, Lyytikäinen O, Sane J (2017). Knowledge, attitudes, and practices regarding ticks and tick-borne diseases, Finland. Ticks Tick Borne Dis.

[24] Namgyal J, Tenzin T, Checkley S (2021). A knowledge, attitudes, and practices study on ticks and tick-borne diseases in cattle among farmers in a selected area of eastern Bhutan. PLoS One.

[25] Cairns G, de Andrade M, MacDonald L (2013). Reputation, relationships, risk communication, and the role of trust in the prevention and control of communicable disease: a review. J Health Commun.

[26] Ophir Y, Jamieson KH (2021). The effects of media narratives about failures and discoveries in science on beliefs about and support for science. Public Underst Sci.

[27] Sellnow TL, Seeger MW (2020). Theorizing Crisis Communication.

[28] Brady RM, Lemieux CJ, Doherty ST (2024). Ticks and lyme disease in natural areas: a segmentation analysis of visitor perceptions of risk and preferred communication strategies. J Outdoor Recreat Tour.

[29] Koinig I, Kohler S (2021). On the relationship between skepticism towards and reactance to health messages: the special case of online communication on tick-borne encephalitis. Front Commun.

[30] Yang CX, Baker LM, McLeod-Morin A (2024). Trending ticks: using Google Trends data to understand tickborne disease prevention. Front Public Health.

[31] Bhattacharjee S, Raj S (2016). Social media: shaping the future of agricultural extension and advisory services. https://www.researchgate.net/publication/330397283_Social_media_Shaping_the_future_of_agricultural_extension_and_advisory_services.

[32] Avidar R, Ariel Y, Malka V, Levy EC (2015). Smartphones, publics, and OPR: do publics want to engage?. Public Relat Rev.

[33] Malecki KMC, Keating JA, Safdar N (2021). Crisis communication and public perception of COVID-19 risk in the era of social media. Clin Infect Dis.

[34] Taylor M, Kent ML (2014). Dialogic engagement: clarifying foundational concepts. J Public Relat Res.

[35] Yang CX, Baker LM, McLeod-Morin A Ticks on X: uncovering sources and engagement rate.

[36] Hwong YL, Oliver C, Van Kranendonk M, Sammut C, Seroussi Y (2017). What makes you tick? The psychology of social media engagement in space science communication. Comput Human Behav.

[37] Pollett S, Rivers C (2020). Social media and the new world of scientific communication during the COVID-19 pandemic. Clin Infect Dis.

[38] Chan MPS, Winneg K, Hawkins L, Farhadloo M, Jamieson KH, Albarracín D (2018). Legacy and social media respectively influence risk perceptions and protective behaviors during emerging health threats: a multi-wave analysis of communications on Zika virus cases. Soc Sci Med.

[39] Gamboa J, Lamb MM, de la Cruz P, Bull S, Olson D (2019). Using social media to increase preventative behaviors against arboviral diseases: a pilot study among teens in the Dominican Republic. Mhealth.

[40] Zhang J, Wu HC, Chen L, Su Y (2022). Effect of social media use on food safety risk perception through risk characteristics: exploring a moderated mediation model among people with different levels of science literacy. Front Psychol.

[41] Tweeten JF, Paulsen TH (2020). Perceptions of communication tools as defined by Iowa cattle producers. NACTA J.

[42] Gillespie JL (2011). U.S. beef producers' current use and perceptions of social media as a communications tool [Master’s thesis]. https://openresearch.okstate.edu/server/api/core/bitstreams/32fc7eca-04b6-4daf-87eb-64a1c834a0e1/content.

[43] Begho T, Glenk K, Anik AR, Eory V (2022). A systematic review of factors that influence farmers’ adoption of sustainable crop farming practices: lessons for sustainable nitrogen management in South Asia. J Sustain Agric Environ.

[44] Ofuoku AU (2012). Influence of extension agents’ and farmers’ communications factors on the effectiveness poultry technology messages. Trop Agric Res Ext.

[45] Seli L, Akbar M, Arianto A (2024). Supporting and inhibiting factors of agricultural extension interpersonal communication competence in Enrekang Regency. KnE eng.

[46] BenYishay A, Mobarak AM (2019). Social learning and incentives for experimentation and communication. Rev Econ Stud.

[47] Lasdun V, Harou AP, Magomba C, Guereña D (2025). Peer learning and technology adoption in a digital farmer-to-farmer network. J Dev Econ.

[48] Entman RM (1993). Framing: toward clarification of a fractured paradigm. J Commun.

[49] Hameleers M (2021). Prospect theory in times of a pandemic: the effects of gain versus loss framing on risky choices and emotional responses during the 2020 coronavirus outbreak – evidence from the US and the Netherlands. Mass Commun Soc.

[50] Sleigh J, Amann J, Schneider M, Vayena E (2021). Qualitative analysis of visual risk communication on Twitter during the COVID-19 pandemic. BMC Public Health.

[51] Tversky A, Kahneman D (1992). Advances in prospect theory: cumulative representation of uncertainty. J Risk Uncertain.

[52] O’Keefe DJ, Jensen JD (2007). The relative persuasiveness of gain-framed and loss-framed messages for encouraging disease prevention behaviors: a meta-analytic review. J Health Commun.

[53] O’Keefe DJ, Jensen JD (2008). Do loss-framed persuasive messages engender greater message processing than do gain-framed messages? A meta-analytic review. Commun Stud.

[54] Gantiva C, Jiménez-Leal W, Urriago-Rayo J (2021). Framing messages to deal with the COVID-19 crisis: the role of loss/gain frames and content. Front Psychol.

[55] Cho H, Boster FJ (2008). Effects of gain versus loss frame antidrug ads on adolescents. J Commun.

[56] Li S, Coduto KD, Song C (2020). Comments vs. one-click reactions: seeking and perceiving social support on social network sites. J Broadcast Electron Media.

[57] Olechnowicz C, Leahy J, Gardner A, Sponarski CC (2023). Perceived vulnerability for Lyme disease questionnaire: a social science tool for understanding tick-borne disease attitudes. Ticks Tick Borne Dis.

[58] Baker R, Brick JM, Bates NA (2013). Summary report of the AAPOR task force on non-probability sampling. J Surv Stat Methodol.

[59] Eysenbach G (2004). Improving the quality of web surveys: the Checklist for Reporting Results of Internet E-Surveys (CHERRIES). J Med Internet Res.

[60] Fake tweet/X generator, Instagram posts generator and many more. Zeoob.

[61] (2024). 2022 Census of Agriculture. National Agricultural Statistics Service United States Department of Agriculture.

[62] Davis JA (1971). Elementary Survey Analysis.

[63] Sumner EM, Ruge-Jones L, Alcorn D (2017). A functional approach to the Facebook Like button: an exploration of meaning, interpersonal functionality, and potential alternative response buttons. New Media & Society.

[64] Alhabash S, Almutairi N, Lou C, Kim W (2019). Pathways to virality: psychophysiological responses preceding likes,shares, comments, and status updates on Facebook. Media Psychol.

[65] Overbey KN, Jaykus LA, Chapman BJ (2017). A systematic review of the use of social media for food safety risk communication. J Food Prot.

[66] Zeballos Rivas DR, Lopez Jaldin ML, Nina Canaviri B, Portugal Escalante LF, Alanes Fernández AMC, Aguilar Ticona JP (2021). Social media exposure, risk perception, preventive behaviors and attitudes during the COVID-19 epidemic in La Paz, Bolivia: a cross sectional study. PLoS One.

